# Antioxidant Response of *Yarrowia lipolytica* Cells: Functional Analysis of Genes Encoding Catalases

**DOI:** 10.3390/jof12040240

**Published:** 2026-03-26

**Authors:** Clara A. Quiñones-González, Maricela Villarreal-García, Miranda Campos-González, Paulette Rascon-Godard, Eduardo Campos-Góngora

**Affiliations:** Universidad Autónoma de Nuevo León, Centro de Investigación en Nutrición y Salud Pública, Facultad de Salud Pública y Nutrición, Av. Dr. Eduardo Aguirre Pequeño y Yuriria, Col. Mitras Centro, Monterrey 64460, Nuevo León, Mexico; claraquigon@hotmail.com (C.A.Q.-G.); maricela.villarrealgr@gmail.com (M.V.-G.); miranda.campos.gzz@gmail.com (M.C.-G.); rascon701@gmail.com (P.R.-G.)

**Keywords:** oxidative stress, reactive oxygen species, cellular antioxidant response, gene expression, catalase, *CAT1*, *CAT2*, *CAT3* genes, *Yarrowia lipolytica*

## Abstract

Oxidative stress (OS) is generated by the imbalance between reactive oxygen species (ROS) and antioxidant enzyme activities, such as catalases, superoxide dismutases, and glutathione peroxidases. In the *Y. lipolytica* genome, three genes encoding catalases (*CAT1*, *CAT2*, and *CAT3*) have been identified; all three genes are transcriptionally active in cells grown under OS conditions. This study aimed to analyze whether the *CAT1* and *CAT2* genes exhibit a compensatory function that allows maintaining the functionality of the antioxidant response in *Y. lipolytica* cells lacking the *CAT3* gene. The construction of the mutant strain (Ylcat3-Δ) was performed using Double-Joint PCR. OS was induced by the addition of H_2_O_2_ [5 mM], ROS production was quantified by fluorescence using 2′,7′-dichlorofluorescein diacetate (DCFH-DA), and gene expression was analyzed by semi-quantitative RT-PCR in both parental (P01a) and mutant (Ylcat3-Δ) strains exposed or not to oxidative conditions. ROS production was lower in P01a cells than in Ylcat3-Δ cells when exposed to H_2_O_2_ [5 mM]. Also, under OS conditions, *CAT1* gene expression levels decreased in both strains, whereas *CAT2* gene expression increased in both types of cells. Under OS, both parental and Ylcat3-Δ strains showed similar growth rate, sensitivity to oxidative conditions and gene expression patterns, and it can be concluded that *CAT3* gene deletion does not alter the transcriptional activity of *CAT1* and *CAT2* genes, suggesting that the compensatory function among the *CAT* genes of *Y. lipolytica* may not be limited to the presence/absence of *CAT3* gene.

## 1. Introduction

Biological processes such as breathing, digesting food, metabolizing xenobiotics, and lipoperoxidation produce byproducts such as superoxide radical (O_2_^•−^), hydrogen peroxide (H_2_O_2_), hydroxyl radicals (^•^OH), and singlet oxygen (^1^O_2_). Such molecules are commonly defined as reactive oxygen species (ROS) [1,2,3,4]. Under physiological conditions, ROS participate in essential cellular signaling pathways; however, when there are ROS accumulation, they can interact with macromolecules (lipids, proteins and nucleic acids) causing cellular damage and contributing to the development of oxidative stress (OS) [5,6,7,8,9]; the lipids of cell membranes can be damaged by an increase in the membrane fluidity and permeability; on the other hand, the protein damage involves the modification of amino acids, peptide chain fragmentation, enzymatic inactivation, electric charge alteration, protein aggregation and proteolysis, and DNA damage involves oxidizing deoxyribose, breaking strand, removing nucleotides, modifying bases, and crosslinking DNA–protein [6]. Free radicals in normal conditions are usually removed by the natural cellular antioxidant system, which is integrated by enzymatic and nonenzymatic molecules. An imbalance between the quantity and activity of these reactive substances and antioxidant mechanisms may cause a condition called oxidative stress (OS), which has been associated with degenerative processes and several diseases [9,10].

In response to OS conditions, organisms activate the synthesis of antioxidant enzymes (catalases, superoxide dismutases, glucose-6-phosphate dehydrogenase, and glutathione peroxidases, among others) and produce metabolites capable of detoxifying ROS [3,11]. Among all antioxidant enzymes, catalases are present in almost all aerobic organisms; they break down hydrogen peroxide into oxygen and water [12]. Increased catalase activity has been reported in yeast cells under OS conditions, suggesting that catalases play a preponderant role in yeast adaptation to OS [2,11,12,13,14,15,16]. Some yeast species possess two or more genes encoding catalase enzymes [17], whereas other species, such as *Candida glabrata,* contain only a single catalase-encoding gene, and it has been demonstrated that the lack of this enzyme (cta1-Δ) showed an alteration in their growth rate under OS conditions [18].

The *Yarrowia lipolytica* genome contains three *CAT* genes (identified by in silico analysis), and we hypothesized that the presence of the three genes encoding catalases allows it to maintain antioxidant activity when one *CAT* gene is lost. Previously, in our laboratory, we analyzed the transcriptional response of antioxidant defense genes in *Y. lipolytica* cells exposed to oxidative conditions induced by H_2_O_2_ [5 mM]. All genes reached maximal expression within the first 15 min; however, *CAT3* and *SOD* showed the highest expression levels, suggesting that the catalase encoded by *CAT3* may play an important role in the antioxidant defense system of this dimorphic yeast [19,20,21]. Therefore, to assess whether the absence of the *CAT3* gene in *Y. lipolytica* modifies the capacity of the antioxidant defense system, we generated a *CAT3* deletion mutant, assessing and evaluating *CAT1* and *CAT2* gene expressions and ROS production in cells subjected to normal and oxidative conditions. This study provides insights into the contribution of catalases to the antioxidant response of *Y. lipolytica* and improves our understanding of genetic redundancy and OS adaptation in yeasts.

## 2. Materials and Methods

### 2.1. Microorganisms and Culture Conditions

The *Y. lipolytica* strain P01a (*MAT A*, *leu* 2-270, *ura* 3-302) was used as a control and as a genetic background for the generation of the mutant strain (Ylcat3-Δ). The *E. coli/pCR2.1-TOPO/URA3* strain [22] was used as a source of the *URA3* gene, which served as a marker gene, for the replacement of the *CAT3* gene ORF in the disruption cassette. *Y. lipolytica* strains were cultured in a liquid or solid YPD medium (1% yeast extract, 2% peptone, 2% glucose, and 2% agar, when required) or a YNB medium (17 g/L) supplemented with glucose (8 g/L), leucine (0.26 g/L), ammonium sulfate (5 g/L), and agar (8 g/L), when required. For experiments under different conditions, yeast cells in the exponential growth phase were inoculated (initial cell density OD_600_ = 0.2) in 250 mL Erlenmeyer flasks containing 50 mL of the YPD liquid medium. The *E. coli/pCR2.1-TOPO/URA3* strain was cultured in the liquid LB medium supplemented with ampicillin (100 μg/mL).

The yeast and bacterial cultures were incubated at 28 °C or 37 °C, respectively, with shaking (200 rpm) overnight.

### 2.2. Construction of Disruption Cassette and Ylcat3-Δ Mutant

**Figure 1:**
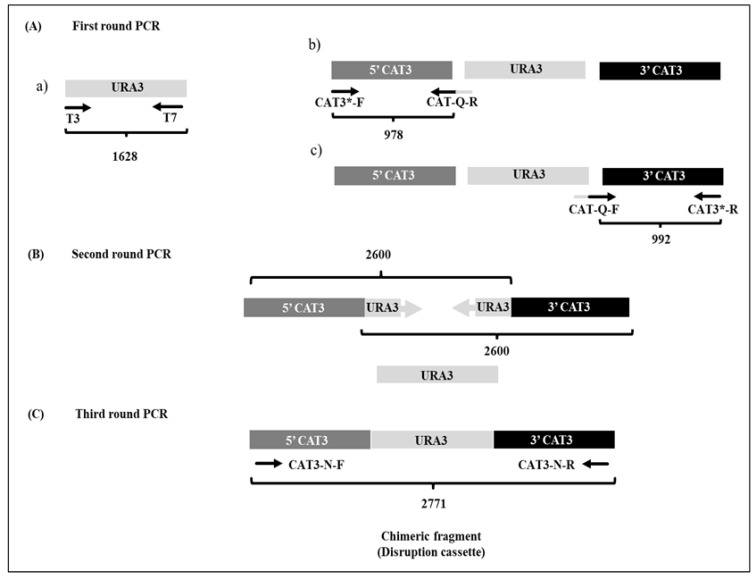
Scheme of the disruption cassette construction. (**A**) First-round PCR: (**a**) amplification of the *URA3* gene marker with universal primers T3 and T7, and amplification of the 5′ and 3′ regions ((**b**) and (**c**), respectively) of the *CAT3* gene with the chimeric and specific primers. (**B**) Second-round PCR: overlap extension PCR with the amplified fragments from the first-round PCR. (**C**) Third-round PCR: amplification of the disruption cassette (*CAT3-URA3-CAT3*) from the products of the second reaction; the disruption cassette was amplified with the nested primers (F and R). In each PCR, the size of the obtained products is represented in base pairs (bp).

**Figure 2:**
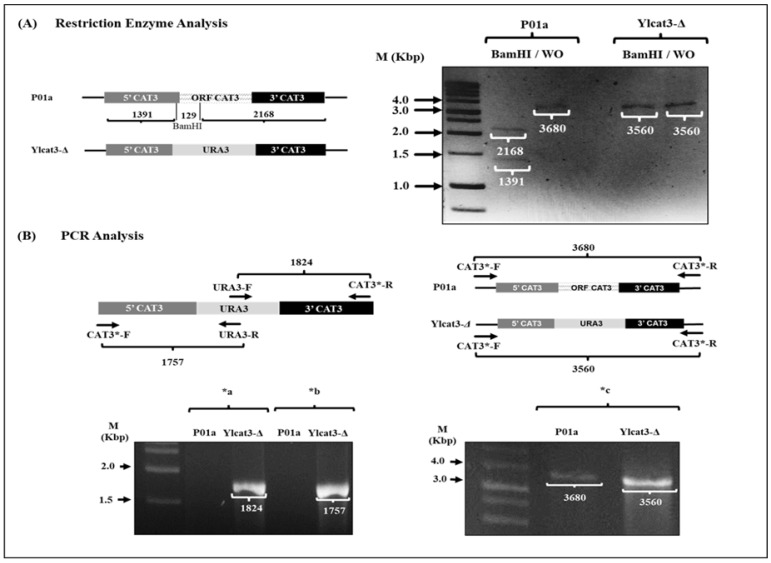
Genotypic analysis of mutant Ylcat3-Δ strain. (**A**) Schematic representation and restriction pattern (BamHI enzyme) in the Ylcat3-Δ and parental strains. Experimental analysis of enzymatic restriction products in the parental (lanes 1 and 2) and mutant (lanes 3 and 4) strains. The molecular weight marker (M; 1 kilobase pairs (Kbp)) was used to identify the size of bands produced by digestion. BamH1/WO: enzymatic digestion with BamH1/without digestion. (**B**) Genotypic analysis by PCR; the *URA3* gene insertion at the locus corresponding to the *CAT3* gene was determined. In bottom (**B**), *a, *b, and *c correspond to PCR products obtained with different primer combinations as indicated at the top of (**B**).

The nucleic acids (DNA and RNA) were isolated from *Y. lipolytica* cells (P01a strain) after overnight culture, using the glass bead lysis protocol described by Hoffman and Winston, which combines chemical and mechanical methods for cellular lysis [23]. The extraction of plasmid DNA (from *E. coli* cultures) was performed by the Birnboim method [24]. The nucleic acids were quantified and stored at −20 °C. The Double-Joint PCR (DJ-PCR) technique [25] was used to amplify the disruption cassette for the transformation of *Y. lipolytica* cells. This procedure was carried out using a set of specific primers/oligonucleotides (Table 1) and Platinum^TM^ Taq DNA Polymerase, High Fidelity (Invitrogen^TM^, Waltham, MA, USA). The DJ-PCR involved three PCRs. In the first PCR (Figure 1A), the 5′ and 3′ flanking regions of the *CAT3* gene were obtained from the genomic DNA of the parental strain by using chimerical primers (**CAT-Q-F**: 5′-GAGAGAGAAGCCAAGATACGTGTGTTAGCGTTGTAGT-3′ or **CAT-Q-R**: 5′-GAGTCAGACATACT-CGTCCTCGGCGTTTCGCTACC-3′) and specific primers (CAT3*-F: 5′-GCTTCCAGTAGTGGCAATATGCGTG-3′ and CAT3*-R: 5′-CATCCTGAGACCATCCTTGTCGG-3′) designed to anneal to the adjacent regions (5′ and 3′) of *CAT3* gene ID: YALI0F30987g. The marker *URA3* gene (1700 bp) was amplified by PCR using the universal primers **T3** and **T7** (Invitrogen^TM^, Waltham, MA, USA) from plasmid DNA obtained from the *E. coli/pCR2.1-TOPO/URA3* strain. In the second PCR (Figure 1B), the products obtained from the first PCR, corresponding to the 5′ and 3′ regions and the *URA3* gene, were used both as primers and templates, respectively. In the third PCR (Figure 1C), the specifically designed “nested primers” (**CAT3-N-F**: 5′-GTCCGTCCT-CGCTCTAACACGTTG-3′ and **CAT3-N-R**: 5′-GGTCTTTCGCTTGGGCTTGATACG-3′) and products from the second PCR were used as primers and the template, respectively. The PCR products were visualized by agarose gel electrophoresis and purified using organic extraction following standard methods [26]. To generate the *Y. lipolytica* mutant cells, the disruption cassette (2771 bp, corresponding to the PCR product amplified with “nested primers”; Figure 1C) was purified and inserted into the genome of lithium-competent *Y. lipolytica* cells by electroporation, following the protocol described by Wang et al. [27]. Mutant cell candidates were selected based on their growth capacity in a selective medium (YNB plus leucine, without uracil). Additionally, restriction enzyme (BamHI) and PCR analyses were performed using primers designed to the ORF of the *URA3* gene and adjacent non-handled regions of the *CAT3* gene (Figure 2).

### 2.3. Oxidative Stress Induction, Cell Growth, Sensitivity and ROS Quantification

The OS induction was achieved by exposure to hydrogen peroxide [5 mM]; to test the effect of *CAT3* gene deletion (mutant strain Ylcat3-Δ) on cell growth, we analyzed and compared its growth (OD_600_) with that of the parental (P01a) strain in the presence or absence of H_2_O_2_ [5 mM] at different times (24–120 h). The cultures in YPD medium were incubated at 28 °C with orbital shaking (200 rpm), and the results were documented by measuring OD_600_ every 24 h until 120 h of incubation.

Additionally, the sensitivity of the mutant strain was compared with that of the parental strain using serial dilutions of cultures grown in YPD and YPD + H_2_O_2_. Both the parental (P01a) and mutant (Ylcat3-Δ) strains were tested at different H_2_O_2_ concentrations [0, 1, 3, 4.5, 5 and 10 mM]. Briefly, using the plate serial dilution spotting method as described by Sherman (2002) [28], 3 μL from each dilution was spotted onto YPD agar plates containing different concentrations of the oxidizing agent and incubated for 24 to 72 h at 28 °C in the dark.

The addition of H_2_O_2_ [5 mM] reduced cell growth, but it did not produce a lethal effect in either strain, as previously reported for the P01a strain [19,29]. To document the results, photographs were taken every 24 h with a photodocumentation system (GelDoc-It Imaging System, UVP).

The ROS quantification was performed using the fluorogenic probe 2,7′-dichlorofluorescein diacetate (DCFH-DA; Sigma-Aldrich, St. Louis, MO, USA). Briefly, *Y. lipolytica* cells in the logarithmic phase corresponding to the parental and mutant strains were cultured under OS conditions (YPD medium supplemented with H_2_O_2_ [5 mM]) at 28 °C for 15 min. After treatment, the culture optical density (OD_600_) was determined and adjusted to 1.0 with MilliQ H_2_O (5 mL final volume); then, cells were collected by centrifugation (12,000× *g*/3 min/TA; microcentrifuge 5415D; Eppendorf^®^, Hamburg, Germany) and exposed to a solution of DMSO/PBS/DCFH-DA [1 mM] or DMSO/PBS (control group, without fluorophore). The cells were incubated for 1 h in the dark, washed twice with PBS, suspended in 1.2 mL PBS buffer, and 200 µL/well was placed into a 96-well plate. The fluorescence was measured using a fluorimeter (Fluoroskan Ascent FL, Thermo Fisher Scientific; Waltham, MA, USA) with excitation/emission wavelengths of 485/538 nm, respectively. Measurements of fluorescence were performed in quadruplicate. The Relative Fluorescence Units (RFUs) were estimated by subtracting the fluorescence value of the control group (DMSO/PBS) from the fluorescence value of the cells treated with DMSO/PBS/DCFH-DA [1 mM].

### 2.4. Nucleic Acid Extraction and mRNA Purification

Nucleic acids were isolated from the *Y. lipolytica* cells (P01a and Ylcat3-Δ mutant strains), as described in Section 2.2, under both oxidative and non-oxidative conditions. To obtain DNA-free RNA for mRNA analysis, DNA was removed from the nucleic acid samples by treatment with DNase I (PureLink, Invitrogen; Waltham, MA, USA), following the manufacturer’s instructions. Briefly, 1000–1500 ng of nucleic acid was incubated with 1 μL (ca. 3 enzymatic units) of the enzyme for 1 h at 37 °C, followed by enzyme inactivation with 1 μL of EDTA [25 mM] at 65 °C for 10 min. The final RNA concentration was determined using a NanoDrop ND-1000 spectrophotometer^TM^ (Thermo Fisher Scientific; Waltham, MA, USA) and adjusted to 100 ng/μL with DEPC–water (diethyl pyrocarbonate–water).

### 2.5. cDNA Synthesis and Semi-Quantitative RT-PCR

cDNA synthesis was performed using the GoScript^TM^ Reverse Transcription System (Promega^®^, Madison, WI, USA) following the manufacturer’s instructions. For RT reactions, 500 ng of purified RNA and oligo dT (0.5 µg) were used. The resulting cDNA was used in semi-quantitative RT-PCR with DNA polymerase (MyTaq, BIOLINE^®^; Alvinston, ON, Canada) and specific primers designed for each analyzed gene (see Table 1). The constitutive *ALG9* gene was used to normalize gene expression levels across all experiments.

### 2.6. Gene Expression Analysis

The transcriptional analysis of the three *CAT* genes was performed in both (parental and mutant) strains using semi-quantitative RT-PCR. Polymerase chain reactions (PCRs) were conducted with a conventional methodology [26], using the cDNA obtained by reverse transcription from the mRNA of cells growing in the logarithmic phase cultured with or without oxidative conditions, DNA polymerase, and specific primers for *CAT1*, *CAT2*, and *CAT3* genes (YALI0E34265g, YALI0E34749g, and YALI0F30987g, respectively), designed to amplify the *CAT* genes of *Y. lipolytica* (Table 1).

Before the sample analysis by RT-PCR, several critical parameters were assessed: the number of PCR cycles (which allows the detection of differences in the expression of genes between the analyzed samples). Furthermore, the amount of cDNA used in each reaction was determined. The different numbers of cycles of PCR (15, 20, 22, 25, and 32 cycles) and different amounts of cDNA (50, 100, 200, 500, and 1000 ng) were tested (Figure S1). As a result of these experimental approaches, the PCRs were performed in a Thermocycler (Sprint Thermal Cycler, Thermo Electron Corporation, Waltham, MA, USA), with 500 ng of cDNA and 22 PCR cycles for the expression analysis of each gene. Each PCR cycle for expression analysis was carried out under the following conditions: denaturation, 95 °C, 30 s; annealing, 60 °C, 60 s; extension, 72 °C, 60 s.

The RT-PCR products were resolved by electrophoresis on 2% agarose gels stained with ethidium bromide and visualized using the GelDoc-It Imaging System UVP analyzer (UVP; Uplan, CA, USA). The size of the amplified fragment was determined by comparison with the molecular weight marker (1500 bp Ladder; Hyperladder IV, BIOLINE^®^, Cincinnati, OH, USA). The bands corresponding to each gene were analyzed by densitometry using the Launch VisionWorks LS software, version number 7.1 (UVP; Uplan, CA, USA). Gene expression levels were calculated for samples under OS and control conditions.

### 2.7. Phylogenetic Tree Construction

A phylogenetic analysis was conducted to determine the putative location of each catalase encoded by the *CAT* genes of *Y. lipolytica*. Multiple sequence alignment and phylogenetic tree construction were performed using the ClustalW program (https://www.genome.jp/tools-bin/clustalw, accesed on 1 April 2025) and MEGA (Molecular Evolutionary Genetics Analysis) software, version 10.1. The nucleotide sequences of genes encoding catalase enzymes from yeasts, including *Saccharomyces cerevisiae*, *Pichia pastoris*, *Candida albicans*, *Candida glabrata*, and *Y. lipolytica*, were obtained from the NCBI (https://www.ncbi.nlm.nih.gov, accesed on 1 April 2025) and KEGG (https://www.genome.jp/kegg/, accesed on 1 April 2025 databases.

### 2.8. Statistical Analysis

All experiments were performed in triplicate or quadruplicate. Data normality was assessed using the Shapiro–Wilk test, and the homogeneity of variances was evaluated using Levene’s test. Intracellular ROS levels following acute H_2_O_2_ exposure (15 min) and *CAT* gene expression levels were compared between strains and treatments. When data presented a normal distribution, statistical comparisons were performed using one-way ANOVA followed by Tukey’s post hoc test, whereas the Kruskal–Wallis test was applied for non-normally distributed data. In addition, repeated-measures ANOVA with Bonferroni correction was used, when appropriate, to evaluate differences in the growth rate between the parental and Ylcat3-Δ strains, both in the presence or absence of H_2_O_2_. Data are presented as mean ± standard deviation or median and interquartile range, as appropriate. Statistical analyses were performed using IBM SPSS Statistics^®^ version 22.0. Statistical significance was set at *p* < 0.05.

## 3. Results

### 3.1. Construction and Analysis of Ylcat3 Mutant

The Ylcat3-Δ strain was constructed with the Double-Joint PCR technique. Table 1 shows the primers used for the generation of mutant cells and for the gene expression analysis. Figure 1 depicts the strategy used for the construction of the disruption cassette, which was inserted into the genome of *Y. lipolytica* cells (for description see Section 2.2).

The genotypic characterization of putative mutant cells was performed using both restriction enzyme and PCR analyses (Figure 2). In silico restriction analysis with the BamH1 enzyme showed two restriction sites in the *CAT3* gene locus (present in the P01a strain), and no restriction sites were found in the disruption cassette inserted in the Ylcat3-Δ strain (with the *CAT3* gene deleted). Experimental analysis (using BamH1) corroborated the absence of the disruption cassette in the P01a strain and the band of the disruption cassette (2771 bp) in the genome of cells from the Ylcat3-Δ strain (Figure 2A). PCR analyses using different combinations of primers (shown at the top of Figure 2B) identified the presence of the disruption cassette containing the marker gene (*URA3*) in the genome of Ylcat3-Δ mutant cells (bottom of Figure 2B) and its absence in cells of the P01a strain.

### 3.2. CAT3 Deletion Does Not Modify Cell Growth or Susceptibility of Y. lipolytica Cells Exposed to Oxidative Conditions

**Figure 3:**
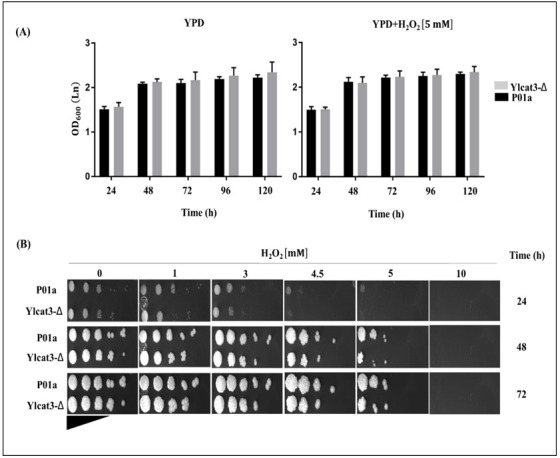
Effect of H_2_O_2_ on the growth and susceptibility of the mutant strain Ylcat3-Δ. (**A**) Comparative growth analysis of the mutant strain Ylcat3-Δ and the parental strain P01a cultured in YPD medium and YPD supplemented with H_2_O_2_ [5 mM] during 24–120 h at 28 °C. Each column represents the mean ± standard deviation of the OD_600_ measurements in cultures of both strains. Statistical analysis was performed using repeated-measures ANOVA with Bonferroni correction. Data represents the average of three independent experiments. (**B**) Comparative analysis of the sensitivity to oxidative conditions of mutant strains Ylcat3-Δ and P01a in the YPD medium supplemented with different concentrations of the oxidizing agent. Cultures of both strains in the logarithmic growth phase (YPD medium, 28 °C, 20 h, 200 rpm) were performed, and serial dilutions (1:10) were prepared. From each culture, OD_600_ was adjusted to 1.0, and 3 µL of each dilution was spotted onto YPD plates with or without H_2_O_2_. Plates were incubated at 28 °C for different times, and photographs were taken every 24 h.

The performed analysis showed that the deletion of the *CAT3* gene does not modify the growth of the mutant cells (strain Ylcat3-Δ) on the YPD medium with or without the addition of the oxidizing agent (Figure 3A); the growth of the mutant strain was similar to that of the P01a strain [19,29]. No significant differences were observed in the growth of both strains (*p* < 0.05) at 24, 48, 72, 96, and 120 h in the presence or the absence of oxidative conditions. The qualitative susceptibility analysis using the plate serial dilutions showed that 10 mM of H_2_O_2_ is lethal for both strains, as no growth was observed after 72 h of incubation; however, both 4.5 and 5 mM H_2_O_2_ showed an inhibitory effect on the growth of both strains (Figure 3B). Based on these results, we decided to use 5 mM of H_2_O_2_ for the remaining experiments in this study.

### 3.3. CAT3 Deletion Modifies the ROS Production in Y. lipolytica Cells Exposed to Oxidative Conditions

**Figure 4:**
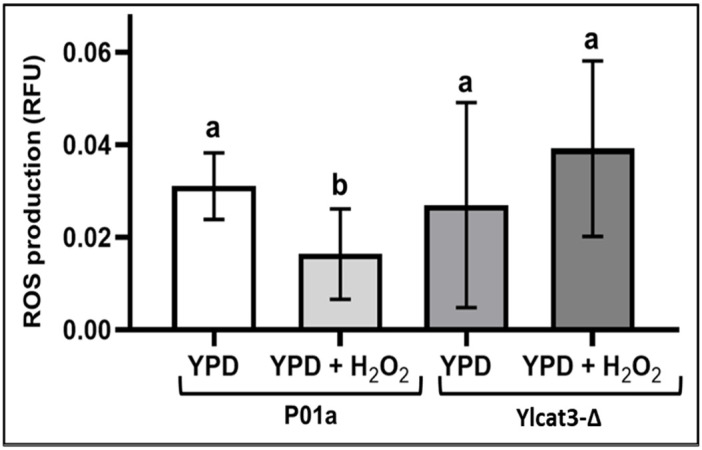
Comparison of ROS production in the P01a and Ylcat3-Δ strains of *Y. lipolytica*, exposed or not to H_2_O_2_ [5 mM]. Data is presented as medians and interquartile ranges from nine replicates across three independent experiments. Different letters indicate significant differences between groups according to the Kruskal–Wallis test (*p* < 0.05). RFU: Relative Fluorescence Units. ROS: reactive oxygen species. P01a: parental strain. Ylcat3-Δ: mutant strain.

ROS levels were analyzed in parental and mutant strains in the presence or absence of H_2_O_2_ [5 mM] (Figure 4). Intracellular ROS levels were determined using the fluorescent probe DCFH-DA. Exposure to H_2_O_2_ significantly decreased ROS production in the parental strain. On the other hand, in the cells of the mutant strain exposed to H_2_O_2_, the ROS production showed an increase compared with their non-exposed counterparts, although the observed differences were not significant, an approximately 2.3-fold increase was observed. Also, comparison between cells (from parental and mutant strains) exposed to oxidative conditions showed a lower ROS production (*p* < 0.05) in the parental cells. For instance, ROS production or accumulation was greater in the cells lacking the *CAT3* gene.

### 3.4. CAT3 Deletion Does Not Modify the Expression Pattern of the Other CAT Genes

**Figure 5:**
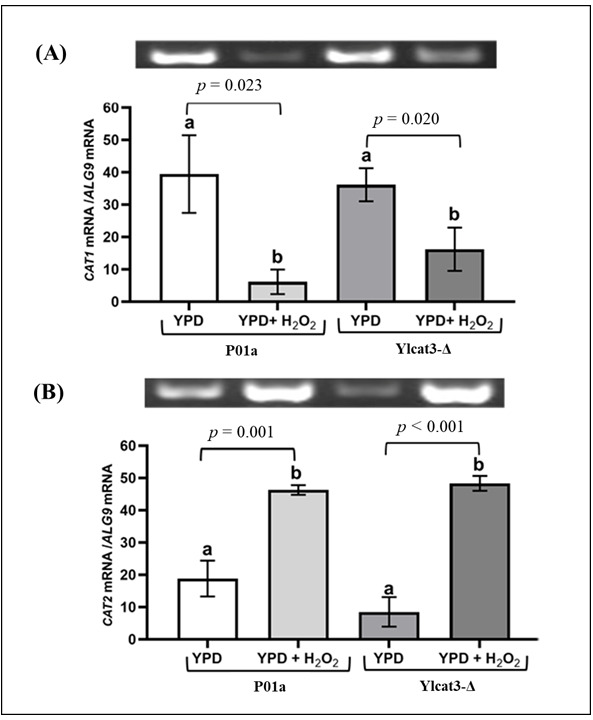
*CAT1* (**A**) and *CAT2* (**B**) gene expression in *Y. lipolytica* cells of parental (P01a) and mutant (Ylcat3-Δ) strains, with and without exposure to H_2_O_2_. Expressed values are the mean of nine replicates from three independent experiments; the bar (at the top of each column) represents the standard deviation (SD). A representative image of the products obtained by RT-PCR in each group is shown at the top of the graph. Different letters indicate statistically significant differences (*p* < 0.05) between groups compared by ANOVA and post hoc Tukey test.

For the gene expression analysis, the RT-PCR products were separated by agarose gel electrophoresis and quantified by densitometry. The changes in the expression levels of the different *CAT* genes in both P01a and Ylcat3-Δ strains were calculated and compared. Both parental and mutant strains in normal conditions (YPD without H_2_O_2_ [5 mM]) showed a higher *CAT1* gene expression than cells exposed to the oxidizing agent. On the other hand, in the presence of H_2_O_2_, *CAT1* gene expression significantly decreased in both parental (*p* = 0.023) and mutant (*p* = 0.020) strains (Figure 5A) compared to non-exposed cells. In contrast, *CAT2* gene expression was higher in P01a (*p* = 0.001) and Ylcat3-Δ (*p* < 0.000) cells treated with H_2_O_2_ than in cells cultured without the oxidant. In both strains, *CAT2* gene expression reached similar levels when cells were exposed to H_2_O_2_ (Figure 5B).

All strains displayed comparable *CAT1* expression patterns in the absence of OS. In the presence of H_2_O_2_, *CAT1* gene expression decreased in both strains. The expression levels of the *CAT2* gene increased in both strains in response to H_2_O_2_, P01a (*p* = 0.003), and Ylcat3-Δ (*p* = 0.011). The *CAT3* gene exhibited a similar pattern to *CAT2*, reaching higher expression levels when cells were exposed to OS conditions. Numeric expression values for each *CAT* gene are shown in Table 2.

### 3.5. Phylogenetic Analysis of Y. lipolytica Catalases

**Figure 6:**
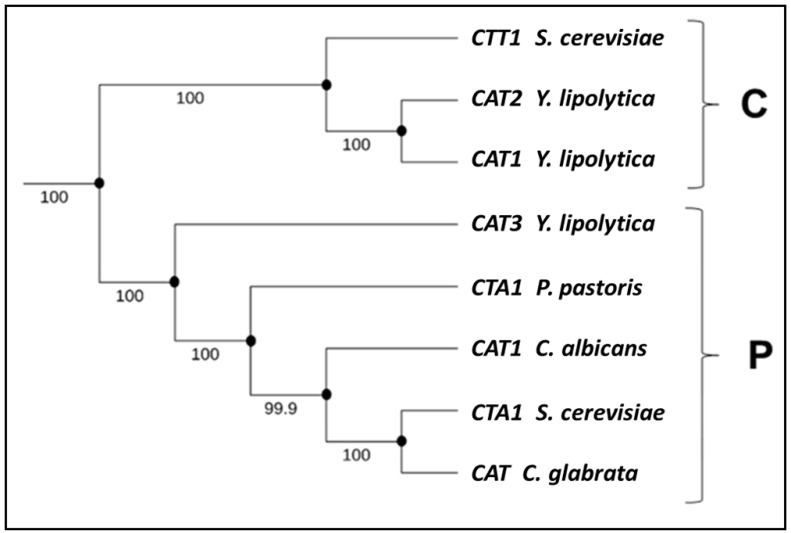
Phylogenetic tree of catalases from different yeast species. For the construction, different gene sequences coding for catalases in yeast species (*S. cerevisiae: Saccharomyces cerevisiae* (*CTT1*: NC_001139.9, *CTA1*: NC_001136.10); *Y. lipolytica: Yarrowia lipolytica* (*CAT1*: YALI0E34265g, *CAT2*: YALI0E34749g, *CAT3*: YALI0F30987g); *P. pastoris: Pichia pastoris* (*CTA1*: AB472085.1); *C. albicans: Candida albicans* (*CAT1*: CAALFM_C106810WA); *C. glabrata: Candida glabrata* (*CTA1*: CAGL0K10868g)) were included. C: cytosolic catalase clade; P: peroxisomal catalase clade. The phylogenetic tree was constructed with MEGA and the ClustalW programs.

A phylogenetic analysis was performed to compare the nucleotide sequences of the genes encoding catalases in *Y. lipolytica* and catalase genes from *S. cerevisiae*, *C. albicans*, *C. glabrata*, and *P. pastoris*. The results revealed the phylogenetic relationship (Figure 6) among the catalase-encoding genes from different analyzed species, showing that the included sequences cluster into two well-defined clades corresponding to cytosolic and peroxisomal catalases.

In *S. cerevisiae*, the catalase Cttp1p, encoded by the *CTT1* gene, is found in the cytosol [30]. Due to the percentage of identity between *CTT1* and sequences corresponding to the *CAT1* and *CAT2* genes of *Y. lipolytica,* we have inferred that the enzymes encoded by these genes are found in the cytosol of *Y. lipolytica* cells (Figure 6).

By contrast, the *CAT3* gene of *Y. lipolytica* clustered on the same branch of the phylogenetic tree as the *CTA1* gene, which encodes peroxisomal catalases in both *S. cerevisiae* and *P. pastoris* [30,31,32], as well as with the sequence of the gene *CAT* encoding catalase in *C. glabrata*, a species reported to possess a single gene coding for this enzyme [18]. Additionally, the *CAT3* gene of *Y. lipolytica* clustered with the *CAT1* gene encoding for a catalase of *C. albicans*, which can be found in both peroxisomes and mitochondria [33].

## 4. Discussion

Reactive oxygen species (ROS) such as superoxide radical (O_2_^•−^), singlet oxygen (^1^O_2_), hydroxyl radical (HO^•^), and hydrogen peroxide (H_2_O_2_) are products of cellular metabolism [34]. The physiological levels of ROS are key in various signaling pathways; however, high ROS concentrations may induce OS, leading to cellular damage at both structural and functional levels [35], which contributes to the development of chronic degenerative pathologies [36]. Catalase activity is one of the most common cellular antioxidant defense mechanisms, observed when cells are exposed to oxidizing agents such as H_2_O_2_ [30,31,32,33,37].

We showed that P01a and Ylcat3-Δ strains presented similar ROS levels without exposure to the oxidizing agent. Izawa, Inoue, and Kimura (1996) [38] reported that catalases in *S. cerevisiae* cells might not be important under normal physiological conditions; however, they become essential under “emergency” or “adaptation” to OS. It has been reported that the cellular localization of catalases (peroxisomal or cytosolic) determines susceptibility to H_2_O_2_; in *S. cerevisiae*, catalases A and T (encoded by the *CTA1* and *CTT1* genes, respectively) can exert compensatory action protecting the cells from OS generated by H_2_O_2_, in response to the absence of catalase genes [38,39,40,41,42]. Consistent with our results, the Ylcat3-Δ strain shows no susceptibility to H_2_O_2_, given that, in the *Y. lipolytica* genome, three genes encoding catalase (*CAT1*, *CAT2*, and *CAT3*) could be involved in a compensatory mechanism among catalases encoded by these genes.

The results obtained with the Ylcat3-Δ strain showed that the deletion of the *CAT3* gene modifies ROS production when cells are exposed to H_2_O_2_-induced oxidative stress. The mutant cells exhibited a notable increase in ROS levels compared with the parental strain (*p* > 0.05). Our analysis indicates that the parental strain (P01a) showed a higher antioxidant response upon exposure to H_2_O_2_, since the ROS values were significantly lower than those generated under normal conditions. Furthermore, our results differ from those reported for *S. cerevisiae* by Izawa et al. [36]. In our system (*Y. lipolytica* cells), catalases encoded by the *CAT1*, *CAT2*, and *CAT3* genes were activated both under OS conditions and in their absence, as observed in the parental strain; however, a different phenomenon was observed in the mutant cells (Ylcat3-Δ), in which ROS production increased upon exposure to the oxidizing agent. These results suggest that, under normal conditions (without OS), the catalase encoded by the *CAT3* gene is responsible for the decreased ROS production observed in P01a cells.

Conversely, the antioxidant response of yeast, including catalase activity, has been reported to be influenced by nutrient availability in the culture medium, and it has been suggested that products derived from glucose metabolism could contribute to the repression of catalase activity [30]. In our study, we used only the YPD medium (which used glucose as a carbon source); based on a previous report, it is possible that glucose and/or its metabolic products may repress catalase expression by inhibiting the transcription of *CAT* genes. In *S. cerevisiae* cells lacking peroxisomal catalase (encoded by the *CTA1* gene), exposure to H_2_O_2_ is not lethal compared to cells lacking cytosolic catalase (encoded by the *CTT1* gene) [38].

The regulation of gene expression under OS conditions in yeast is more complex than in prokaryotes, as it has been reported that the expression of at least 450 genes is required to maintain cellular resistance to ROS [32,34]. Genetic redundancy is a common feature of living organisms; their genomes have a large proportion (15 to 65%) of duplicated genes [42,43,44]. Duplicate genes have the potential for acquiring novel functions; however, not all gene duplication events result in functional innovation. Notably, about 25% of duplicate genes in yeast are from the whole-genome duplication and encode metabolic enzymes [44,45].

It has been demonstrated, in model organisms, that complete gene inactivation typically has little or no phenotypic effect, mainly due to two mechanisms responsible for such a functional compensation of mutations: genetic redundancy is one of these mechanisms, whereby the deletion of one gene has little effect due to the presence of its duplicate and functionally overlapping paralogous gene [46]. The second mechanism relies on the distributed nature of genetic networks; interactions among genes with unrelated functions also provide functional compensation [47,48]. It has been reported that only the double null mutant of *S. cerevisiae* lacking both Cta1 and Ctt1 is sensitive to OS, whereas single mutants show no stress-related phenotype [38]. These findings suggest that these activities are functionally redundant, which can be explained by the gene duplication that encodes for catalases in this species.

The phylogenetic analysis showed evidence suggesting that the *CAT1* and *CAT2* genes of *Y. lipolytica* encode putative cytosolic catalases, whereas the *CAT3* gene encodes a putative peroxisomal catalase. Based on these observations, it is plausible that, as reported for *S. cerevisiae* [30], *P. pastoris* [32], *C. glabrata* [18], and *C. albicans* [33], the *CAT3* gene or peroxisomal catalase deletion in *Y. lipolytica* cells does not represent a significant defense mechanism against OS generated by H_2_O_2_ addition. In support of the above, we observed that *CAT3* gene deletion in *Y. lipolytica* cells did not lead to transcriptional changes in the other *CAT* genes (*CAT1* and *CAT2*) when cells were exposed to H_2_O_2_. In addition, it is important to mention that the genome of *Y. lipolytica* contains three genes encoding for catalases (*CAT1*, *CAT2* and *CAT3*); also, another series of genes that are part of this antioxidant response have been identified: one gene encoding superoxide dismutase enzyme (*SOD* gene), one gene encoding for a copper chaperone for Sod (*CCS* gene) and one gene encoding for the enzyme glutathione peroxidase (*GPX* gene). Each of these genes is transcriptionally active in response to OS conditions [21] and may contribute to the compensatory mechanism proposed here.

## 5. Conclusions

The *CAT3* gene deletion in *Y. lipolytica* cells did not induce changes in the expression levels of *CAT1* and *CAT2* genes when exposed to H_2_O_2_ [5 mM]. These results suggest that, contrary to the hypothesis of this study, the deletion of *CAT3* gene in *Y. lipolytica* cells did not modify the expression of *CAT1* and *CAT2* genes, and there is no compensatory mechanism of the antioxidant response mediated by *CAT* genes. However, a slight increase in ROS production was observed when mutant cells were exposed to H_2_O_2_.

The parental strain (P01a) showed a greater antioxidant response upon exposure to H_2_O_2_, which was not observed in the mutant strain (cells lacking the *CAT3* gene), suggesting that the catalase encoded by the *CAT3* gene is responsible for the decreased ROS production observed in P01a cells.

Consistent with phylogenetic analysis, the *CAT3* gene of *Y. lipolytica* is predicted to encode a peroxisomal catalase, whereas *CAT1* and *CAT2* are predicted to encode cytosolic catalases. To our knowledge, this study provides one of the first descriptions of *CAT* gene expression in *Y. lipolytica* cells under oxidative conditions.

## Figures and Tables

**Table 1:** Oligonucleotides used in this work.

Oligonucleotide Name	Oligonucleotide Sequence (5′ → 3′)	Used for
**ALG9**	**F:** CCGGCGACTTTGCGATACTGTGCC **R:** CCAGCAACAGCAATGAGCACAAAGCC	Gene expression (Constitutive gene-data normalization)
**CAT1**	**F:** CCACCACCGTGCGATTTTCTACC **R:** CATGGTCTGAAGGGAAACGGTCC	Gene expression (Determination)
**CAT2**	**F:** CCATGCAAAGGGAGGAGGAGCC **R**: CCGTCCACGAGGGGTAATCCC	Gene expression (Determination)
**CAT3**	**F**: CAAGACCTTCACTCGATTCTCCACC **R**: CGTCATTGGTGAGGTTCTTGATGCC	Gene expression (Determination)
CAT3*	**F**: GCTTCCAGTAGTGGCAATATGCGTG **R**: CATCCTGAGACCATCCTTGTCGG	Specific primers for *CAT3* gene; amplify *CAT3* ends (1st round DJ-PCR). Corroborate the correct insertion of the disruption cassette
**CAT-Q**	**F**: **GAGAGAGAAGCCAAGATAC**GTGTGTT- AGCGTTGTAGT **R**: GAGTCAGACAGATACTC**GTCCTCGGC-** **GTTTCGCTACC**	Chimeric primers; amplify *CAT3* ends (1st round DJ-PCR).
**T3, T7**	**T3:** GCAATTAACCCTCACTAAAGG **T7:** TAATACGACTCACTATAGGG	Universal primers; amplify the marker gene (*URA3*) from plasmidic DNA
**CAT3-N**	**F**: GTCCGTCCTCGCTCTAACACGTTG **R**: GGTCTTTCGCTTGGGCTTGATACG	Nested primers; obtain the disruption cassette (3rd round DJ-PCR)
**URA3**	**F**: GGCCTGCGAGCTGGTGCCGAGG **R**: CCTCGGCACCAGCTCGCAGGCC	*URA3* internal primers; analyze the correct insertion of the disruption cassette in the *Y. lipolytica* genome

F: forward primer; R: reverse primer; ALG9, CAT1, CAT2, and CAT3: specific primers for *ALG9*, *CAT1*, *CAT2*, and *CAT3* genes, respectively; CAT-Q: chimeric primers, the underline/bold letters correspond to nucleotides from the *URA3* gene; CAT3-N: nested primers, designed on the sequences corresponding to the 5′ and 3′ ends of the *CAT3* gene; URA3: internal primers, designed on the sequence of the *URA3* gene.

**Table 2:** Expression levels of *CAT* genes in *Y. lipolytica* with or without OS conditions.

Strain		P01a		Ylcat3-Δ	
Treatment		YPD	YPD + H_2_O_2_	YPD	YPD + H_2_O_2_
**Gene**	*CAT1*	34.7 ± 12.7	7.3 ± 3.9	34.8 ± 5.2	16.3 ± 6.7
	*CAT2*	19.5 ± 5.6	46.3 ± 1.5	10.5 ± 4.9	47.3 ± 2.5
	*CAT3*	12.1 ± 3.5	61.9 ± 5.5	ND	ND

Data corresponds to the mean ± standard deviation obtained from triplicate samples from three independent experiments. ND = not detected.

## Data Availability

The original contributions presented in this study are included in the article/Supplementary Material. Further inquiries can be directed to the corresponding author.

## Supplementary Materials

The following supporting information can be downloaded at: https://www.mdpi.com/article/10.3390/jof12040240/s1, Figure S1: Standardization of parameters for semi-quantitative RT-PCR. (A) Nucleic acids (DNA and RNA) extracted from *Y. lipolytica* cells using the Hoffman and Winston (1987) protocol [23]; quality was assessed by agarose gel electrophoresis. (B) PCR amplification of nucleic acid samples subjected to different DNase treatments (Tx); genomic DNA (gDNA) was included as a positive PCR control (+). Amplifications were performed using specific CAT2 primers. All DNase Tx. were effective for gDNA removal. (C) Different cDNA concentrations were tested in PCR reactions after the RT step using specific primers for *CAT2* gene; PCR product intensity increased with increasing cDNA concentration. (D) Optimization of PCR cycle number using different cDNA quantities. The figure shows representative results obtained with 22 and 30 PCR cycles. Using 22 cycles, clear differences in amplification intensity were observed depending on the amount of cDNA used as template. RT, reverse transcription; PCR, polymerase chain reaction; DNA, deoxyribonucleic acid; RNA, ribonucleic acid; Tx, treatments; DNase, deoxyribonuclease; gDNA, genomic DNA; CAT2, Y. lipolytica CAT2 gene; cDNA, complementary DNA synthesized from mRNA by RT. Figure S2: RT-PCR analysis of catalase genes in *Y. lipolytica* parental (P01a) and mutant (Ylcat3-Δ) strains under H_2_O_2_ stress. Products were amplified from cDNA obtained by RT of parental or mutant cells using forward and reverse primers specific for each gene (described in Table 1). The absence of *CAT3* gene amplification in Ylcat3-Δ cells, whether exposed or not to oxidative conditions, confirms that the mutant strain lacks this gene. Numbers correspond to the expected fragment size (bp). YPD: cultures in YPD medium; YPD + H_2_O_2_: cultures in YPD supplemented with H_2_O_2_ [5 mM].
