# Supplementary material for: Antioxidant Response of Yarrowia lipolytica Cells: Functional Analysis of Genes Encoding Catalases
Source: J Fungi (Basel). 2026 Mar 26;12(4):240. doi: 10.3390/jof12040240 (PMC13117495; doi:10.3390/jof12040240)
Supplement: Supplementary file 1 [file jof-12-00240-s001.zip › jof-4173933-supplementary.pdf]

Suppl. Figure S1.

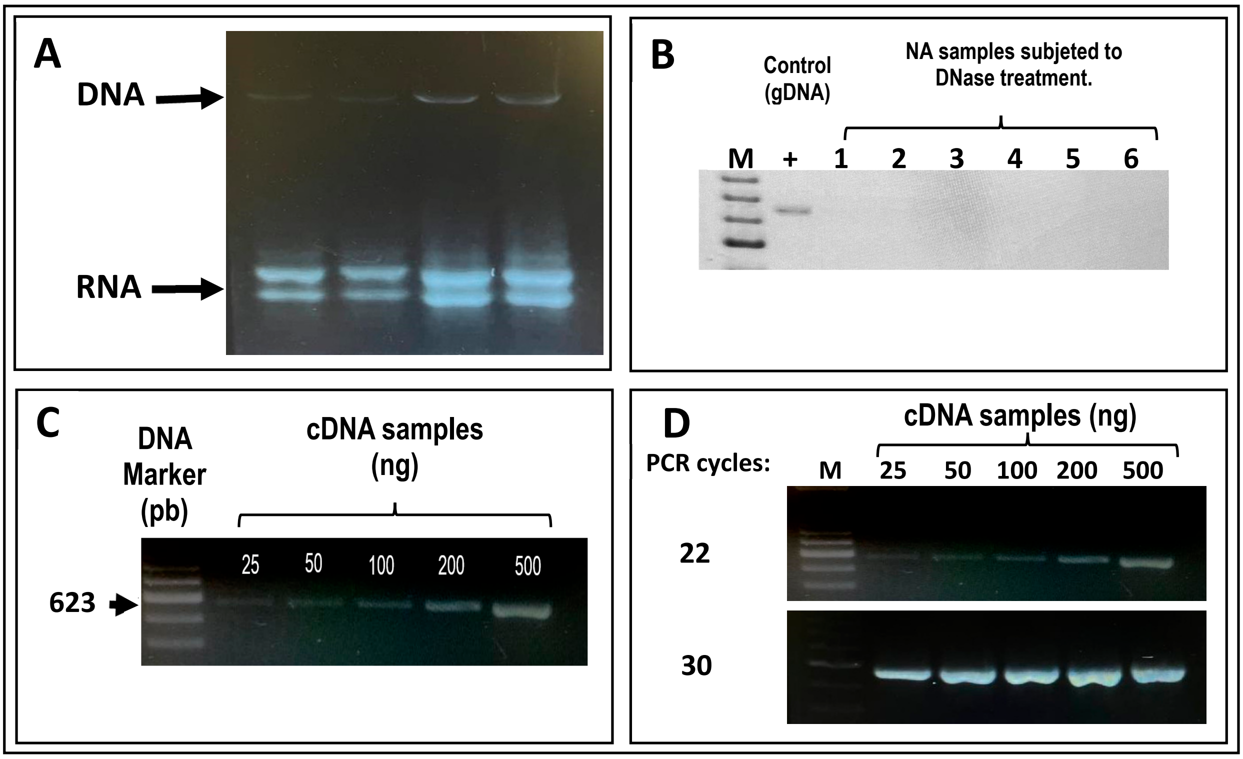

Figure S1. Standardization of parameters for semi-quantitative RT-PCR. (A) Nucleic acids (DNA and RNA) extracted from *Y. lipolytica* cells using the Hoffman and Winston (1987) protocol; quality was assessed by agarose gel electrophoresis. (B) PCR amplification of nucleic acid samples subjected to different DNase treatments (Tx); genomic DNA (gDNA) was included as a positive PCR control (+). Amplifications were performed using specific CAT2 primers. All DNase Tx. were effective for gDNA removal. (C) Different cDNA concentrations were tested in PCR reactions after the RT step using specific primers for *CAT2* gene; PCR product intensity increased with increasing cDNA concentration. (D) Optimization of PCR cycle number using different cDNA quantities. The figure shows representative results obtained with 22 and 30 PCR cycles. Using 22 cycles, clear differences in amplification intensity were observed depending on the amount of cDNA used as template. RT, reverse transcription; PCR, polymerase chain reaction; DNA, deoxyribonucleic acid; RNA, ribonucleic acid; Tx, treatments; DNase, deoxyribonuclease; gDNA, genomic DNA; CAT2, Y. lipolytica CAT2 gene; cDNA, complementary DNA synthesized from mRNA by RT.

Suppl. Figure S2.

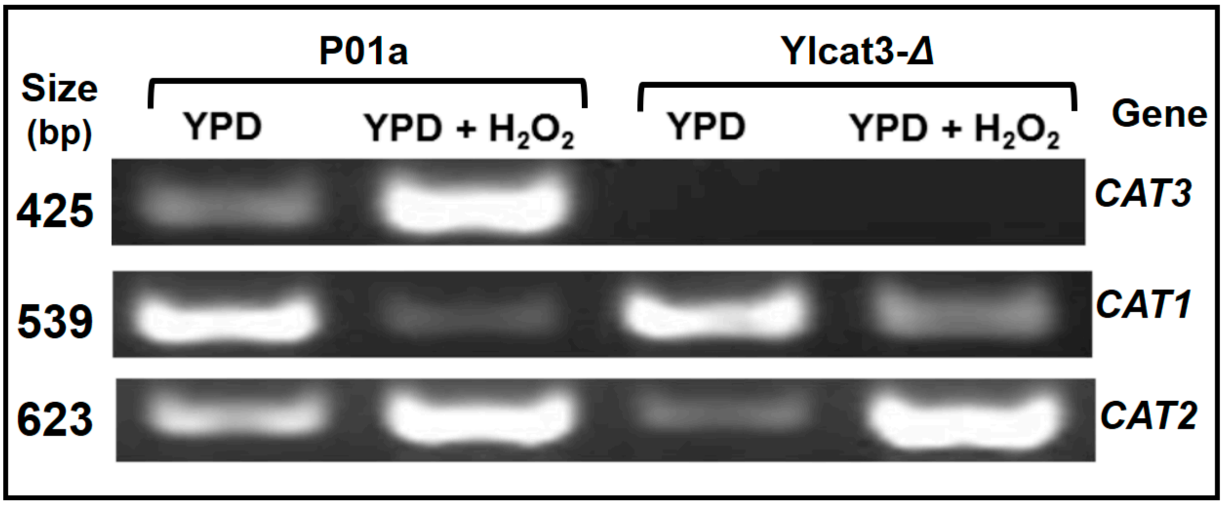

Figure S2. RT-PCR analysis of catalase genes in *Y. lipolytica* parental (P01a) and mutant (Ylcat3-*Δ*) strains under H_2O_2 stress. Products were amplified from cDNA obtained by RT of parental or mutant cells using forward and reverse primers specific for each gene (described in Table 1). The absence of *CAT3* gene amplification in Ylcat3-*Δ* cells, whether exposed or not to oxidative conditions, confirms that the mutant strain lacks this gene. Numbers correspond to the expected fragment size (bp). YPD: cultures in YPD medium; YPD + H_2O_2: cultures in YPD supplemented with H_2O_2 [5 mM].
